# A Comparison of Intermittent and Continuous Exercise Bouts at Different Intensities on Appetite and Postprandial Metabolic Responses in Healthy Men

**DOI:** 10.3390/nu12082370

**Published:** 2020-08-07

**Authors:** Lewis R Mattin, Victoria J McIver, Adora Mo Wah Yau, Lewis J James, Gethin H Evans

**Affiliations:** 1 Department of Life Sciences, Manchester Metropolitan University, Manchester M1 5GD, UK; L.Mattin@mmu.ac.uk (L.R.M.); victoria.mciver@northumbria.ac.uk (V.J.M.); A.Yau@mmu.ac.uk (A.M.W.Y.); 2 Department of Sport, Exercise and Rehabilitation, Northumbria University, Newcastle-Upon-Tyne NE1 8ST, UK; 3 National Centre for Sport and Exercise Medicine, East Midlands, School of Sport, Exercise and Health Sciences, Loughborough University, Loughborough LE11 3TU, UK; L.James@lboro.ac.uk; 4 Department of Sport and Exercise Sciences, Manchester Metropolitan University, Manchester M1 5GD, UK

**Keywords:** exercise modality, gastric emptying rate, appetite, energy intake

## Abstract

Exercise intensity affects many potential postprandial responses, but there is limited information on the influence of exercise modality. Therefore, the aim of this study was to investigate if the nature of exercise at two different intensities would affect gastric emptying rate (GER), appetite and metabolic responses following ingestion of a semi-solid meal. Twelve healthy men completed, in a random order, four 60-min cycles at 60% VO_2peak_ (MOD), 40% VO_2peak_ (LOW) and in a continuous (CON) or intermittent (INT) manner. INT consisted of 20 × 1-min exercise bouts with 2-min rest breaks. INT and CON were matched for total work output at each intensity. GER of the post-exercise meal was measured for 2 h using the 13C-breath method. Blood glucose, substrate utilisation and appetite ratings were measured at regular intervals throughout all trials and 24-h energy intake (EI) post-trials was assessed. GER-Delta over Baseline (DOB) was lower (*p* < 0.05) on MOD-INT vs. MOD-CON from 30–120 min post-meal. Blood glucose was higher mid-exercise (*p* < 0.05) on MOD-INT vs. MOD-CON. Although post-exercise LOW-CON was significantly higher than LOW-INT (*p* < 0.05), blood glucose was also higher 30-min post-meal ingestion on both CON trials compared to INT (*p* < 0.001). No interaction effect was observed for perceived appetite responses 2 h after meal ingestion (all *p* > 0.05). 24-h post-trial EI was similar between LOW-CON vs. LOW-INT (*p* > 0.05), although MOD-INT vs. MOD-CON 3500 ± 1419 vs. 2556 ± 989 kCal: *p* < 0.001 was elevated. In summary, MOD-INT exercise delays GER without stimulating perceived appetite in the 2 h period after meal ingestion, although EI was greater in the 24-h post-trial.

## 1. Introduction

The global prevalence of obesity has dramatically increased, with obesity becoming a leading health concern [1] and contributing to approximately 2.8 million deaths each year world-wide [2]. Obesity is typically the result of a chronic long-term imbalance between energy intake and energy expenditure [3]. Despite health organisations, such as the World Health Organisation (WHO), emphasizing the importance of physical activity, the number of inactive adults continues to rise [4]. Numerous investigations have focused on reducing or stabilising the increase in bodyweight seen from inactivity. Therefore, understanding how physical activity affects the ‘appetite response’ post-exercise is critical.

Moderate intensity continuous exercise (MICE) is the preferred exercise type for recreational activities [5] and favoured during acute energy deficit research [6,7,8,9], which entails exercising at an intensity between 50–65% VO_2 max_ for ≥ 30 min. A recent study by our laboratory displayed that continuous exercise at 70% VO_2Peak_ and 40% VO_2Peak_ resulted in no difference in post-exercise appetite response to a semi-sold meal [10]. This suggests that higher intensity continuous exercise may benefit weight loss goals due to the increased energy expenditure (EE) induced, at least for the same duration of exercise. Whilst the effects of continuous exercise on post-exercise appetite/metabolism has been relatively well documented, less is known about the effects of intermittent exercise in this regard.

In recent years, much research has focused on the effectiveness of intermittent exercise or high intensity intermittent exercise (HIIE), which has been reported to improve physical fitness and cardiovascular disease risk [11,12]. Additionally, some evidence, but not all, suggests intermittent exercise might facilitate greater weight loss [13] than continuous endurance exercise, possibly related to a greater suppression of appetite post-exercise [14,15,16]. Sim et al. [17] found performing three high intensity intermittent exercise sessions per week for 12 weeks improved appetite regulation leading to reduced energy intake (EI) compared to an equivalent period of moderate intensity continuous exercise and control. This suggests long-term benefits of training in an intermittent manner that might increase weight loss. Additional work from this group found when a single exercise session were matched for total workload, EI remained lower after high intensity intermittent exercise compared with moderate intensity continuous exercise as EI was attenuated in the post-exercise meal [18]. Despite showing minor changes in EI, intermittent exercise results in additional physiological adaptation, such as improved muscle oxidative capacity [19,20], that may lead to greater health benefits than continuous exercise.

The effect of exercise intensity on substrate oxidation has been well documented, as predominantly at lower exercise intensities (~40% VO_2 max_) fat oxidation provides energy to perform external work with the remaining energy demand being supplied by carbohydrate oxidation [21,22,23]. The energy demands in the recovery period must be considered [24], as any increase in fat oxidation during exercise may be mitigated by consuming food post-exercise. Horton et al. [25] indicated overfeeding with carbohydrate results in greater 24 h carbohydrate oxidation and energy expenditure whereas fat overfeeding led to minimal changes in fat oxidation and energy expenditure. How individuals metabolise energy post-exercise may have critical long-term consequences for the way in which they store and utilise body fat. Nevertheless, regular overfeeding occurs post-exercise in research studies as a large proportion of the literature uses ad libitum meals [3,26,27] and energy intake can be as high as ~5500 kJ [28]. King et al. [26] provided participants with an ad libitum meal after exercising continually for 50-min at approximately 70% of HR_max_ and failed to demonstrate any effect on appetite nor EI within the same day or on following rest days.

Furthermore, only a small number of studies have examined the relationship between gastric emptying rate (GER) and changes in appetite following acute exercise [7,26,27], and studies that have investigated the impact of exercise on appetite and EI have not included GER as a primary measurement. What we do know is GER may be a rate-limiting step in the delivery of nutrients to the small intestine and an important understudied factor in appetite control [29]. Evans et al. [30] established that exercise intensity appears to have little effect on GER of a glucose solution after 30-min moderate intensity continuous exercise at 33% peak power output (PPO) and a high intensity intermittent exercise bout (10 × 1 min at PPO). Although evidence has suggested that GER of liquids is reduced during high intensity continuous exercise [31,32] and intermittent exercise [33,34], it is currently unclear whether intermittent exercise at different intensities differentially affects GER or stimulates a compensatory increase in appetite response. We do, however, expect the same response in GER post-exercise as what has been found previously during intermittent exercise.

To our knowledge, no investigation has compared the postprandial responses to a single bout of intermittent or continuous exercise at a moderate and low intensity to assess if the modality of exercise is as important as intensity. Therefore, the purpose of this study was to compare intermittent and continuous exercise at a low and moderate intensity to determine whether the type of exercise has an effect on GER leading to a difference in metabolic responses, rating of appetite and 24-h EI following ingestion of a standardised semi-solid meal. It was hypothesised that intermittent exercise regardless of intensity would delay GER and lead to a greater suppression of appetite post-exercise than continuous exercise.

## 2. Materials and Methods

### 2.1. Participants

Fourteen healthy men who matched the criteria (age = 18–40 years; body mass index = < 29.9 kg/m^2^; non-smokers, no history of gastrointestinal symptoms or disease, not taking any prescription medication, had no other relevant medical conditions assessed by a medical screening questionnaire and were habitually physically active) were recruited from central Manchester, UK (Table 1). Verbal and written explanations of the experimental procedures were provided before the start of the trials and written informed consent to participate was obtained. Two participants withdrew prior to completing the study, meaning twelve participants completed the study. An a priori calculation was conducted using previous data from our laboratory which used a similar semi-sold meal [10]. An effect size η^2^*p* = 0.135 from a repeated measures ANOVA model, attributing GER as the primary outcome measure, and using an α of 0.05 and a statistical power of 0.8 determined that ≥ 12 participants would be required to reject the null hypothesis (G*Power 3.0.10, Heinrich Hein Universitat, Dusseldorf, Düsseldorf, Germany). Ethical approval was provided via the Manchester Metropolitan University Faculty of Science and Engineering Research Ethics and Governance Committee (Reference Number: SE1617159).

### 2.2. Preliminary Trial

Participants visited the laboratory on one occasion to undergo exercise testing and anthropometric measurements and were familiarised with the protocol techniques. Questionnaires for health screening, physical activity and dietary habits were completed. Height was measured to the nearest 0.1 cm using a wall-mounted stadiometer and body mass to the nearest 0.01 kg using electronic scales (GFK 150; Adam Equipment Co. Ltd., Milton Keynes, UK). Body fat percentage was estimated using bioelectrical impedance analysis (Bodystat 1500, Body Composition Technology, Isle of Man, UK). Participants were then familiarised with the gastric emptying assessment technique and visual analogue scale (VAS) to be used during the experimental trials.

Peak oxygen uptake (VO_2Peak_) was measured on a cycle ergometer (Lode Excalibur Sport, Groningen, The Netherlands). Expired air was continuously collected using a breath-by-breath gas analyser (Metalyzer 3b, Cortex, Leipzig, Germany). Workload began at 50 W and a cadence of 70 rpm was maintained throughout. Workload was then increased by increments of 50 W every 3 min until respiratory exchange ratio (calculated as VCO_2_/VO_2_) was greater than 1.0 for at least 1 min. From this point onwards, increments of 20 W were applied every minute until volitional exhaustion. VO_2Peak_ was calculated by averaging the oxygen volume consumed over the final 1 min period. Off-line analysis was used to calculate required workloads during the main trials (40 and 60% VO_2Peak_) Exercise intensities were matched for power output by calculating V02 (mL/kg/min) at the highest data point situated on a linear trend line plotted against exercise intensity (W). This established Watt intensity for the continue LOW/MOD trials was used to determined energy expenditure KJ using the equation (Watt’s × time (s) = Joule/1000 = KJ), using the notion (1 W = 1 joule per second). This total energy expenditure (KJ) for the 60 min continuous trials were used to calculate 1 min watt intensity for the intermittent trials. This ensured equal power output between LOW and MOD conditions. Heart rate (HR) was measured continuously using a HR monitor (Polar H9, Kemple, Finland). Before leaving the laboratory, participants were provided with food scales (Salter, ARC 1066 Electronic Kitchen scale Max 3 kg, Tonbridge, UK) and a physical activity and food diary.

### 2.3. Pre-Trial Standardisation

Each experimental trial was preceded by 24-h weighed food and drink intake and an activity maintenance period during which participants were asked to record their diet and activity before the first trial, and then replicate these patterns prior to the next three trials. To ensure that participants were adhering to the dietary standardisation procedures, the research team contacted participants via telephone the day before each main trial. The purpose of this was to ensure standardisation and consistency of macronutrient intake and metabolic status in the 24 h leading to each trial. Furthermore, participants were asked to refrain from alcohol and caffeine consumption and strenuous physical activity in the 24 h preceding each experimental trial.

### 2.4. Experimental Protocol

Experimental trials were conducted in a randomised-crossover design commencing between 0700 and 0800 following an overnight fast from 22:00, with the exception of plain water consumption. Ninety minutes prior to arrival at the laboratory, participants were asked to drink 500 mL of plain water to ensure an adequate hydration status. Experimental trials were separated by a minimum of 7 days and laboratory atmospheric temperature was ~21 °C and a relative humidity of ~30% throughout the trials.

Upon arrival at the laboratory, participants’ post-void body mass was obtained before they completed seated rest for 10 min, where a VAS questionnaire was completed. Further baseline measures of a capillary blood sample and expired gas samples (for calculation of substrate utilisation) were collected for 15 min in a semi-supine position on a bed. Participants wore the mask for 15 min only during resting conditions. Average VO_2_ and VCO_2_ measurements from the last 5 min were used to calculate fat and carbohydrate oxidation rates using stoichiometric equations [35]. This sampling method for expired gas was adhered to for all other expired gas samples at 135, 165, 195 and 225 min to assess postprandial oxidation every 30-min during recovery.

Participants completed a 60 min exercise protocol either at 60% VO_2peak_ (MOD) or 40% VO_2peak_ (LOW) in a continuous (CON) or intermittent (INT) manner. CON consisted of 60-min continuous cycling and INT consisted of 20 × 1 min exercise bouts interspersed by 2 min rest periods. INT and CON at each intensity were matched for total work output. HR and ratings of perceived exertion were recorded every 5 min in CON and at the end of each 1-min exercise bout and 2-min rest period in INT. A capillary blood sample was collected 30 min after the start of the exercise protocol (mid-exercise). During exercise, each participant was provided with a standardized amount of water (125 mL) at 15, 30, 45 and 60 min resulting in a total of 500 mL during the exercise period. After completion of the exercise bout, a further VAS was completed and a capillary blood sample was collected (post-exercise). Participants were given 30 min to shower and change their clothes before food was provided. A further collection of capillary blood and VAS was collected (pre-meal). Participants consumed a standardised meal after which they rested in the laboratory for 2 h.

### 2.5. Meal

The meal consisted of 800 g Heinz classic vegetable soup heated in a microwave. The meal provided 376 kCal/1584 kJ and the macronutrient content was 6.4 g fat, 8.8 g protein, 66.4 g carbohydrates, 7.2 g fibre and 4.8 g salt. Participants had 15 min to consume the standardised meal and the time taken to eat it was recorded. After the meal, no more food or drink was consumed until participants left the laboratory.

### 2.6. Participants’ Appetite Response

Participants rated their fullness, hunger, prospective food consumption (PFC), satisfaction, nausea and bloatedness on horizontal lines 100 mm in length anchored with “Not at all full” to “totally full,” “I am not hungry at all” to “I have never been more hungry,” “Nothing at all” to “a lot,” “I am completely empty” to “I can’t eat another bite,” “Not at all nauseous” to “Very nauseous” and “Not at all bloated” to “Very Bloated” at 0 mm and 100 mm, respectively. VASs were measured at baseline, 60 min (post-exercise), 90 min (pre-meal), immediately post meal (105-min), then every 15 min post meal up to 2 h.

### 2.7. Measurement of Gastric Emptying

GER was assessed using the non-invasive ^13^C-acetate breath method. Meals contained 100 mg ^13^C-sodium acetate (^1–13^C, 99%; Cambridge Isotope Laboratories Inc., Andover MA, USA), which was first dissolved in 20 mL of water before being added to the heated soup. A basal end expiratory breath sample was collected prior to food ingestion (pre-meal) and further breath samples were collected at 15 min intervals for 2 h post ingestion of the test meal (120, 135, 150, 165, 180, 195, 210 and 225-min). Samples were analysed by non-dispersive IR spectroscopy (IRIS Dynamic, Kibion, Germany) for the ratio of ^13^CO_2_:^12^CO_2_. The difference in the ratio of ^13^CO_2_:^12^CO_2_ from baseline breath to post-ingestion breath samples were expressed as delta over baseline (DOB). Half emptying time (T_1/2_) and time of maximum emptying rate (T_lag_) were calculated using the manufacturer’s integrated software evaluation.

### 2.8. Blood Sampling and Analysis

All blood samples were collected using the capillary method, with the participants in a seated position. Capillary blood was taken from the fingertip using a 23 G gauge single use safety lancet (Unistik-3, Owen Mumford, Oxford, UK) and measured for glucose concentration using an automated desk top analyser (Hemocue Glucose 201^+^ analyser, Ângelholm, Sweden). Samples were taken at 0 min (baseline), 30 min (mid-exercise), 60 min (post-exercise), 90 min (pre-meal) and at 30 min intervals post meal ingestion up to 2 h (135, 165, 195 and 225-min).

### 2.9. Post-Trial Energy Intake

Participants completed a weighed record of all food and drink consumed after leaving the laboratory and recorded their diet through to 1300 h the next day for 24 h. Food records were analysed by a member of the research team using the weight documented for each food or ingredient by using manufacturer values provided when possible or by using DietPlan dietary analysis (Software 6, Forestfield software limited, Horsham, West Sussex, UK). This information was used to estimate total energy intake during the 24 h immediately following each trial.

### 2.10. Statistical Analysis

Data were analysed using SPSS version 24 (IBM, New York, NY, USA). Area under the curve (AUC) values were calculated using the trapezoidal method. All data were checked for normality of distribution using the Shapiro–Wilk test. Pre-trial body mass, exercise and environmental measurements, time to eat soup, AUC calculations, gastric emptying T_lag_ and T_1/2,_ and EI were analysed using a two-way repeated measures analysis of variance (ANOVA) for intensity and modality of exercise. Substrate utilisation, DOB, blood glucose, and appetite were analysed using a three-way repeated measures ANOVA for time, intensity and modality of exercise. Sphericity for repeated measures was assessed and Greenhouse–Geisser epsilons were used to correct for violations. Significant main effects were followed by paired student’s *t*-Test or one-way repeated measures ANOVA with Bonferroni adjusted pairwise comparisons as appropriate. Statistical significance was accepted at the 5% level and results are presented as mean ± standard deviation (SD) unless otherwise stated. For all analyses of variance (ANOVA), effect size was calculated as partial eta squared (*η*^2^ *p*). For pairwise comparisons, effect size was calculated as Cohen’s (d) and 95% confidence intervals (CI) are stated. The effect sizes (d) can be interpreted as trivial (<0.20), small (0.20–0.49), moderate (0.50–0.79) or large (≥0.80) [36].

## 3. Results

### 3.1. Exercise and Meal Measurements

Pre-trial body mass was not significantly different between trials with statistical analysis showing no effect of intensity (*p* = 0.256, η^2^*p* = 0.116), modality (*p* = 0.388, η^2^*p* = 0.068) and intensity × modality interaction effect (*p* = 0.726, η^2^*p* = 0.012).

There was no difference in environment temperature between trials with statistical analysis showing no effect of intensity (*p* = 0.262, η^2^*p* = 0.113; *p* = 0.061, η^2^*p* = 0.283), modality (*p* = 0.592, η^2^*p* = 0.027; *p* = 0.793, η^2^*p* = 0.007) and intensity × modality interaction effect (*p* = 0.297, η^2^*p* = 0.097; *p* = 0.752, η^2^*p* = 0.009).

No main effect of modality (*p* = 387, η^2^*p* = 0.019) was shown for average heart rate (HR) during the 60 min exercise period, however a main effect of intensity (*p* < 0.001, η^2^*p* = 0.969) and an intensity × modality interaction (*p* < 0.001, η^2^*p* = 0.582) was observed. Post-hoc tests revealed HR was significantly higher between MOD-CON compared to LOW-CON (139 ± 18 vs. 104 ± 16 bpm: *p* < 0.001, d = 2.15, 95% CI = −8.04–11.20 bpm) and MOD-INT compared to LOW-INT was reported (130 ± 17 vs. 106 ± 16 bpm: *p* < 0.001, d = 1.52, 95% CI = −8.10–10.57 bpm).

No main effect of modality (*p* = 0.118, η^2^*p* = 0.207) nor intensity × modality interaction (*p* = 119, η^2^*p* = 0.150) was shown for total estimated work completed during exercise (KJ), however a main effect of intensity (*p* < 0.001, η^2^*p* = 0.975) was observed. Post-hoc tests revealed significantly more work was completed between LOW-CON compared to MOD-CON (277± 94 vs. 524 ± 104 KJ: *p* < 0.001, d = 2.60, 95% CI = −56.24–55.79 W) and LOW-INT compared to MOD-INT was reported (271 ± 73 vs. 518 ± 103 KJ: *p* < 0.001, d = 2.89, 95% CI = −55.39–44.19 KJ).

Exercise intensity (W) was significantly different between all trials, with statistical analysis demonstrating an effect of intensity (*p* < 0.001, η^2^*p* = 0.944), modality (*p* < 0.001, η^2^*p* = 0.963) and an intensity × modality interaction (*p* < 0.001, η^2^*p* = 0.0957. Post-hoc tests revealed significantly lower exercise intensity for LOW-CON compared to LOW-INT (77± 26 vs. 222 ± 73 W: *p* < 0.001, d = 2.76, 95% CI = −38.54–17.47 W) and MOD-CON compared to MOD-INT was reported (145 ± 29 vs. 423 ± 86 W: *p* < 0.001, d = 4.52, 95% CI = −44.13–20.93 W).

Finally, differences in time to eat the soup were found between trial modality (*p* = 0.005, η^2^*p* = 0.519) but not for intensity (*p* = 0.253, η^2^*p* = 0.117) nor an intensity x modality interaction (*p* = 0.999, η^2^*p* = 0.010). Post-hoc tests revealed time to eat soup was significantly longer during MON-INT compared to MOD-CON (434± 91 vs. 405 ± 101 s: *p* = 0.011, d = 0.32, 95% CI = −51.95–57.32 s) but not between LOW-CON and LOW-INT (313 ± 166 vs. 343 ± 91 s: *p* = 0.456, d = 0.23, 95% CI = −51.25–94.16 s) shown in Table 2.

### 3.2. Appetite Responses

**Figure 1:**
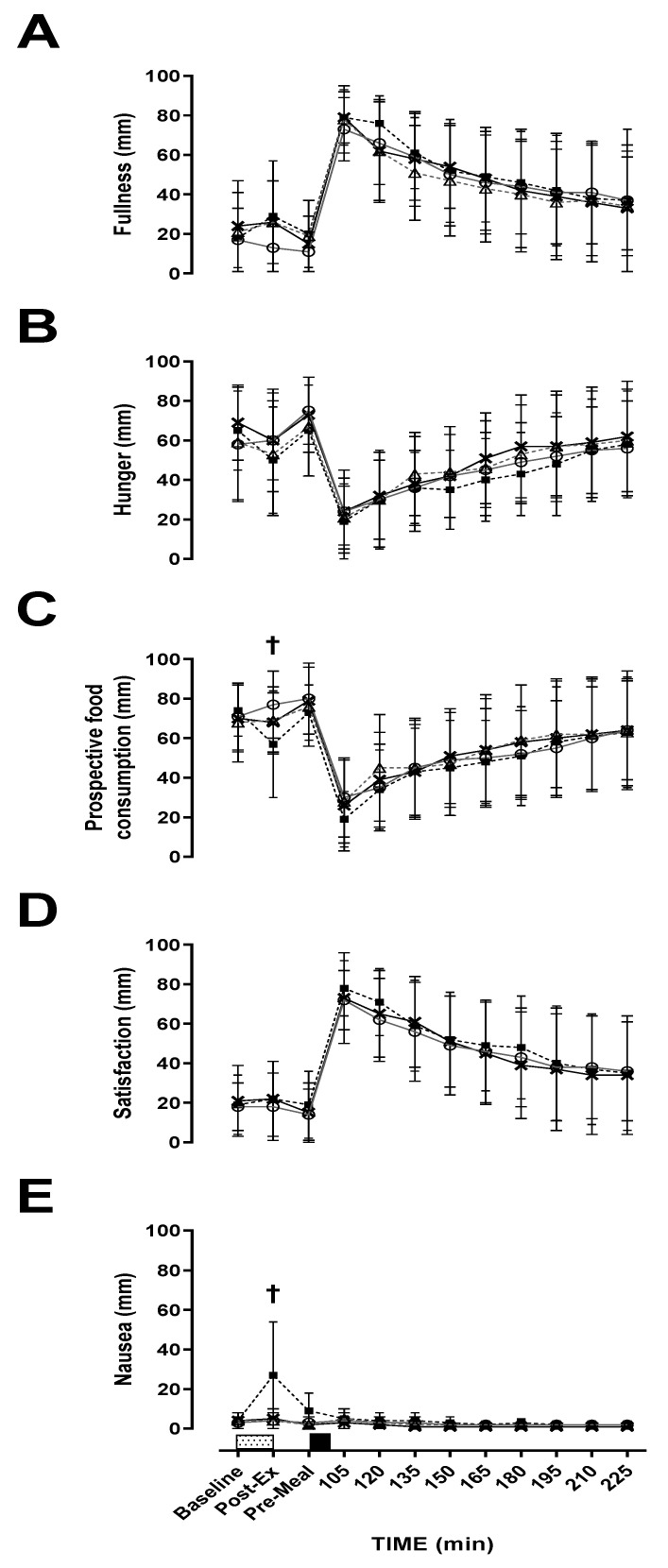
Visual analogue scale (VAS). (**A**) fullness, (**B**) hunger, (**C**) prospective food consumption, (**D**) satisfaction and (**E**) nausea during; LOW-INT (▲), LOW-CON (**○**), MOD-INT (■) and MOD-CON (x). Solid lines represent continuous trials and hashed lines represent intermittent trials. Data points are means with vertical error bars representing SDs (*n* = 12). Unfilled rectangles with black spots indicate 60-min exercise period; filled rectangle indicates a semi-solid meal. **†** Indicates MOD-INT trial significantly different from all other trials, determined by Bonferroni adjusted paired *t*-test (*p* < 0.05).

A main effect of time was observed for fullness (*p* < 0.001, η^2^*p* = 0.617: Figure 1A), hunger (*p* < 0.001, η^2^*p* = 0.510: Figure 1B), PFC (*p* < 0.001, η^2^*p* = 0.534: Figure 1C), satisfaction (*p* < 0.001, η^2^*p* = 0.640: Figure 1D), nausea (*p* < 0.001, η^2^*p* = 0.107: Figure 1E) and bloating (*p* < 0.001, η^2^*p* = 0.373). Hunger and PFC decreased, whilst fullness, satisfaction and bloating increased, whereas nausea was relatively unchanged after food ingestion. There were no intensity or modality effects for hunger (*p* = 0.222; η^2^*p* = 0.132: *p* = 0.895, η^2^*p* = 0.002), fullness (*p* = 0.437, η^2^*p* = 0.056; *p* = 0.237, η^2^*p* = 0.125), PFC (*p* = 0.300, η^2^*p* = 0.097; *p* = 0.485, η^2^*p* = 0.045), satisfaction (*p* = 0.353, η^2^*p* = 0.079; *p* = 0.530, η^2^*p* = 0.037) and bloating (*p* = 0.222, η^2^*p* = 0.076; *p* = 0.895, η^2^*p* = 0.075). There was a main effect of intensity for nausea but not modality (*p* = 0.018, η^2^*p* = 0.413; *p* = 0.069, η^2^*p* = 0.270). Post-hoc tests revealed no further differences between trials for nausea. There was no intensity x modality x time interaction effect observed for hunger (*p* = 0.387, η^2^*p* = 0.976), satisfaction (*p* = 0.430, η^2^*p* = 0.082), or bloating (*p* = 0.47, η^2^*p* = 0.100). However, fullness (*p* = 0.041, η^2^*p* = 0.201), PFC (*p* = 0.04, η^2^*p* = 0.147) and nausea (*p* < 0.001, η^2^*p* = 0.377) did show an interaction. MOD-INT was significantly lower post-exercise compared to the other three trials for PFC (*p* = 0.011, d = 0.54, 95% CI = −8.18–15.84 mm) and higher post-exercise for nausea (*p* = 0.011, d = 1.24, 95% CI = −13.47–4.18 mm). However, post-hoc tests revealed no further differences between trials for fullness.

### 3.3. Gastric Emptying

**Figure 2:**
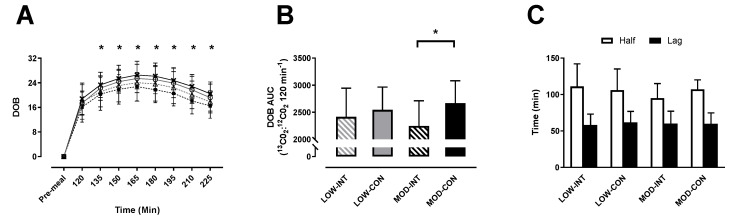
(**A**) Delta over baseline, (**B**) delta over baseline area under curve (AUC), (**C**) half emptying time (T_1/2_) and time of maximum emptying rate (T_lag_). On delta over baseline (DOB) the trials are indicated as; LOW-INT (▲), LOW-CON (**○**), MOD-INT (■) and MOD-CON (x). Solid lines represent continuous trials and hashed lines represent intermittent trials. Data points are means with vertical error bars representing SDs. DOB-AUC bar charts represent mean AUC response (0–120 min) to a standardised meal, with vertical error bars representing SDs. T_1/2_ and T_lag_ data points are means with vertical error bars representing SDs. Unfilled rectangle indicates T_1/2_; black rectangle indicates T_lag_ (*n* = 12). * Indicates MOD-INT values are significantly different than MOD-CON, determined by Bonferroni adjusted paired *t*-test (*p* < 0.05).

No main effect of modality (*p* = 0.760, η^2^*p* = 0.009) or intensity × modality x time interaction (*p* = 0.302, η^2^*p* = 0.711) was observed for delta over baseline. A main effect of time (*p* <0.001, η^2^*p* = 0.987) and intensity (*p* = 0.003, η^2^*p* = 0.568) was observed. All trials and times points were significantly increased from baseline (*p* < 0.001) (Figure 2A).

No main effect of intensity (*p* = 0.745, η^2^*p* = 0.010) was shown for delta over baseline AUC, however a main effect of modality (*p* = 0.003, η^2^*p* = 0.560) and intensity × modality interaction (*p* = 0.041, η^2^*p* = 0.328) were observed. Post-hoc test revealed significantly lower AUC for MOD-INT compared to MOD-CON (2246 ± 467 vs. 2670 ± 412 ^13^CO_2_:^12^CO_2_ 120 min^−1^: *p* = 0.002, d = 1.01, 95% CI = −232.10–265.23 120 min^−1^) (Figure 2B).

No main effect of intensity (*p* = 0.581, η^2^*p* = 0.029), modality (*p* = 0.990, η^2^*p* = 0.011) or intensity × modality interaction (*p* = 0.595, η^2^*p* = 0.027) was observed for T_lag_. There was also no main effect of intensity (*p* = 0.591, η^2^*p* = 0.027), modality (*p* = 0.262, η^2^*p* = 0.113) or intensity x modality interaction effect (*p* = 0.055, η^2^*p* = 0.259; Figure 2C) observed for T_1/2_.

### 3.4. Blood Glucose Concentration

**Figure 3:**
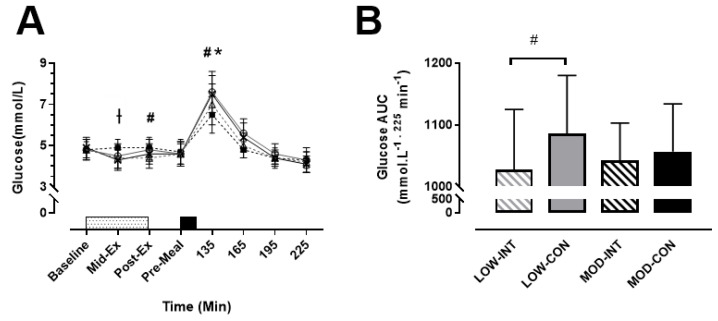
(**A**) Blood glucose, during the LOW-INT (▲), LOW-CON (**○**), MOD-INT (■) and MOD-CON (x). Solid lines represent continuous trials and hashed lines represent intermittent trials. Data points are means with vertical error bars representing SDs (*n* = 12). Unfilled rectangle with black spots indicates 60-min exercise period; filled rectangle indicates a semi-solid meal. (**B**) Bar charts represent mean blood glucose AUC response (0–225 min) to a standardised meal after exercise, with vertical error bars representing SDs. † Indicates MOD-INT trial significantly higher than all other trials. # Indicates LOW-INT are significantly different than LOW-CON. * Indicates MOD-INT are significantly different than MOD-CON, determined by Bonferroni adjusted paired *t*-test (*p* < 0.05).

No main effect of modality (*p* = 0.638, η^2^*p* = 0.021) was observed, however a main effect of intensity (*p* = 0.036, η^2^*p* = 0.342), time (*p* < 0.001, η^2^*p* = 0.882) and intensity x modality x time interaction (*p* = 0.009, η^2^*p* = 0.210) was observed for blood glucose concentration. Post-hoc tests revealed that MOD-INT was significantly higher mid-exercise (4.9 ± 0.4 mmol/L: *p* = 0.016, η^2^*p* = 0.266) compared to the other three trials. LOW-CON was significantly higher than LOW-INT post-exercise (4.8 ± 0.5 vs. 4.4 ± 0.5 mmol/L: *p* = 0.004, d = 0.84, 95% CI = 0.55–1.12 mmol/L). Subsequently, blood glucose was higher 30-min post-meal ingestion for LOW-CON compared to LOW-INT (7.6 ± 1.0 vs. 7.0 ± 1.0 mmol/L: *p* < 0.001, d = 0.64, 95% CI = 0.07–1.20 mmol/L). This was also replicated during the MOD trials as blood glucose was higher during MOD-CON compared to MOD-INT (7.5 ± 0.9 vs. 6.5 ± 0.9 mmol/L: *p* < 0.001, d = 1.07, 95% CI = 0.55–1.57 mmol/L) (Figure 3A).

No main effect of intensity (*p* = 0.634, η^2^*p* = 0.021) nor modality (*p* = 0.107, η^2^*p* = 0.164) was detected, however a main effect of intensity × modality (*p* = 0.022, η^2^*p* = 0.394) was observed for blood glucose AUC. A significantly lower AUC for LOW-INT compared to LOW-CON was reported (1027 ± 96 vs. 1085 ± 93 mmol/L^−1^ 225 min^−1^: *p* = 0.003, d = 0.64, 95% CI = −51.98–54.96 225 min^−1^) (Figure 3B).

### 3.5. Substrate Utilisation

**Figure 4:**
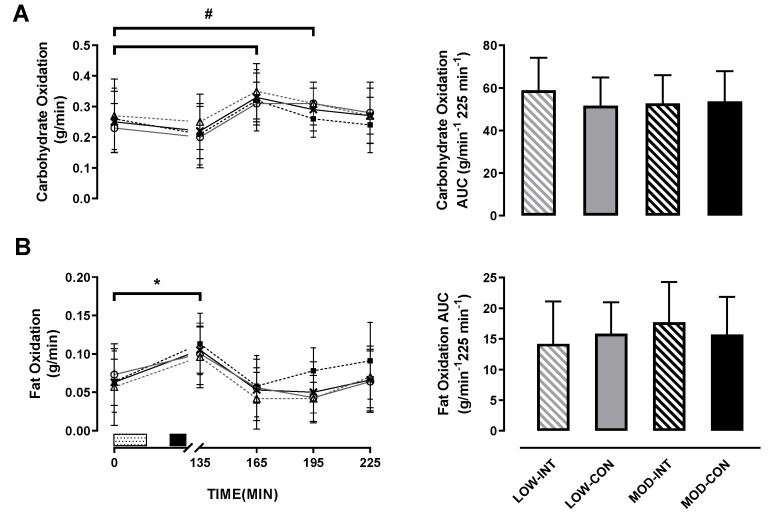
(**A**) Carbohydrate oxidation, and (**B**) fat oxidation during the LOW-INT (▲), LOW-CON (○), MOD-INT (■) and MOD-CON (x). Solid lines represent continuous trials and hashed lines represent intermittent trials. Data points are means with vertical error bars representing SDs (*n* = 12). Unfilled rectangle with black spots indicates 60-min exercise period; filled rectangle indicates ingestion of a semi-solid meal; 135, 165, 195 and 225-min represent post-meal measurements. Bar charts represent mean AUC responses (0–225 min) to a standardised meal after exercise, with vertical error bars representing SDs. # Indicates LOW- CON trials significantly increased from baseline to 60 and 90-min post-meal. * Indicates MOD-INT and CON trials significantly increased from baseline to 30-min post-meal determined by Bonferroni adjusted paired *t*-test (*p* < 0.05).

No main effect of intensity (*p* = 0.677, η^2^*p* = 0.016), modality (*p* = 0.346, η^2^*p* = 0.081), or intensity × modality x time interaction (*p* = 0.766, η^2^*p* = 0.023) was observed for carbohydrate oxidation response. A main effect of time (*p* < 0.001, η^2^*p* = 0.409) was observed (Figure 4A).

There was no main effect of intensity (*p* = 0.543*,* η^2^*p* = 0.035), modality (*p* = 0.445, η^2^*p* = 0.054) nor intensity x modality interaction (*p* = 0.158, η^2^*p* = 0.172) for carbohydrate utilisation AUC.

No main effect for intensity (*p* = 0.639, η^2^*p* = 0.021), modality (*p* = 0.170, η^2^*p* = 0.164) or intensity × modality × time interaction (*p* = 0.939, η^2^*p* = 0.018) was observed for fat oxidation response. A main effect of time (*p* < 0.001, η^2^*p* = 0.512) was observed (Figure 4B).

There was no main effect of intensity (*p* = 0.262*,* η^2^*p* = 0.113) or modality (*p* = 0.921, η^2^*p* = 0.001). An intensity × modality interaction was observed for fat utilisation AUC (*p* = 0.041, η^2^*p* = 0.327; in Figure 4B), however, post-hoc tests revealed no further differences between trials for fat utilisation AUC.

### 3.6. Energy and Macronutrient Intake

**Figure 5:**
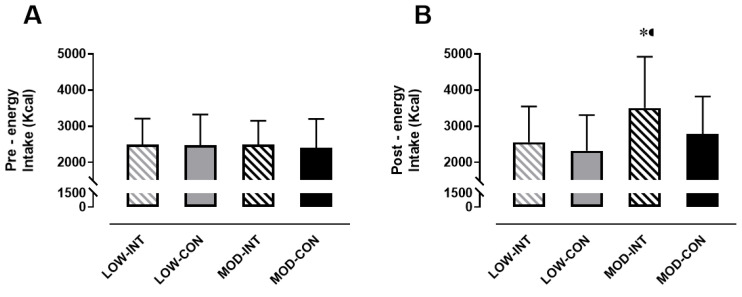
(**A**) Pre-trial energy intake (kCal) (*n* = 12), and (**B**) post-trial energy intake over 24 h (kCal) (*n* = 11). Data points are means with vertical error bars representing SDs. * Indicates MOD-INT is significantly different than MOD-CON, determined by Bonferroni adjusted paired *t*-test (*p* < 0.05).

Pre-trial energy intake amounted to 2483 ± 721 kCal, 2474 ± 854 kCal, 2486 ± 660 kCal and 2396 ± 803 kCal during the LOW-INT, LOW-CON, MOD-INT and MOD-CON trials, respectively, and there was no significant effect for intensity (*p* = 0.745, η^2^*p* = 0.010), modality (*p* = 0.621, η^2^*p* = 0.023) nor intensity x modality (*p* = 0.665, η^2^*p* = 0.018; Figure 5A).

No main effect of the intensity × modality (*p* = 0.093, η^2^*p* = 0.256) interaction was detected for 24 h post trial energy intake (kCal), however a main effect of intensity (*p* = 0.005, η^2^*p* = 0.556) and modality (*p* = 0.001, η^2^*p* = 0.667) was observed. Post-hoc tests revealed MOD-INT was significantly higher compared to MOD-CON (3500 ± 1419 vs. 2777 ± 1042 kCal: *p* < 0.001, d = 0.61, 95% CI = −802.25–590.16 kCal) but no difference was found between LOW-INT and LOW-CON (2556 ± 989 v 2320 ± 985 kCal: *p* = 0.258, d = 0.25, 95% CI = −559.32–557.56 kCal) (Figure 5B).

## 4. Discussion

The primary aim of this investigation was to examine the effect of INT/CON exercise at different exercise intensities on gastrointestinal responses and subsequent appetite response following ingestion of a standardised semi-solid meal. The main findings were that modality of exercise appears to have little impact on the markers measured during this study when performed at a low intensity. However, at a moderate intensity, intermittent exercise delayed GER of a test meal without promoting an acute appetite response in the short 2 h monitoring period after exercise. Nevertheless, 24-h post-exercise EI increased by approximately ~21% in the MOD-INT compared to MOD-CON despite the activities being matched for power output. Further studies are required to determine whether the delay in GER influenced the increase in 24-h EI post-exercise. It is also important to add that the participants in the present study were healthy physically active men, so whether these finding extend to different populations (females or overweight/obese) is currently unknown.

To our knowledge, this is the first study to examine GER of a semi-solid meal after moderate intensity intermittent exercise (MIIE) compared to an energy matched continuous exercise bout. There are a number of physiological factors that regulate GER, including gastrointestinal hormones such as ghrelin, peptide YY (PYY) and glucagon-like peptide-1 (GLP-1). GLP-1 and PYY have been shown to increase post-exercise which may increase parasympathetic activation, providing a potential mechanism as to why GER was delayed in the present study [37]. In contrast, ghrelin levels are usually high pre-exercise and decline immediately after exercise before gradually increasing prior to food intake [10]. Although appetite regulating hormones were not measured within the present study, appetite was assessed using a visual analogue scale (VAS). Previous studies that have used VAS have reported exercise intensity > 60% VO_2max_ results in suppression of appetite in untrained individuals [27,38,39]. In the present study, PFC was significantly lower post-exercise and nausea was higher only during the MOD-INT trial. An increase in nausea within the present study may suggest why subjective measures of hunger were subdued in all trials apart from LOW-CON which increased immediately post-exercise, however, there was no significant difference observed. It is important to add; regardless of this result, hunger in the short 2-h monitoring period after consuming a standardised semi-sold meal responded similarly in recovery, irrespective of modality of exercise or intensity. These findings are consistent with Holliday et al. [40], who reported no significant reduction in subjective appetite when participants completed a bout of high intensity aerobic exercise. Although subjective appetite was unchanged, the time taken to eat the semi-sold meal was marginally longer during the MOD-INT trial compared to MOD-CON. It should be considered that when participants were challenged to consume the whole semi-sold meal 30 min after exercise, it took 29 s longer to consume the meal on the MOD-INT trial compared to its counterpart. This result may suggest a possible suppression in appetite post-exercise indicated by the prospective food consumption data as food volume, energy density and macronutrient composition all influence postprandial fullness [41,42].

Despite appetite responding similarly within the postprandial period in the current study, EI was ~21% (723 kCal) higher 24-h post-trial for MOD-INT compared to MOD-CON. In contrast, the majority of the available research suggests that exercise does not stimulate any changes in energy intake > 20-h after exercise [26,43] when using a self-reported measurement of food intake. In addition, King et al. [27] provided each participant with an overnight food bag and also found energy intake remained unchanged. Intermittent exercise has been suggested to evoke greater weight loss than traditional endurance exercise due to greater reductions in appetite during the post-exercise period [14,16,44]. These findings indicate that the moderate intensity intermittent trial stimulated an increase in EI 24-h post-trial. The mechanism for this increase in EI after the MOD-INT trial is not directly clear. Hengist et al. [45] assessed the metabolic responses to maximal eating and discovers participants who consumed on average nearly double the energy intake when compared to ad libitum eating, had marginal differences in physiological responses and glycaemic control within the post-prandial period, suggesting the increase in EI documented in the current study undoubtedly had very small physiological effects after a one-off single bout of intermittent exercise. However, it must be considered that consuming excess energy will eventually lead to weight gain and therefore increase the risk of developing obesity. When comparing the present findings to previous data, it has been demonstrated among the majority of the scientific literature that land-based exercise does not stimulate increases in energy intake in the hours after exercise [27,44]. It must be noted, examining EI via a weighed dietary assessment may cause recall bias, as the nature of any documentation data collection method has potentially high participant variation. For this reason, caution must be used when interpreting this data.

It has been well documented that during exercise a carbohydrate-electrolyte drink delays gastric emptying and more so during high intensity intermittent exercise [33]. Interestingly, Evans et al. [30] observed that gastric emptying of a 5% glucose solution was not affected by exercise intensity, and therefore GER of a carbohydrate solution was not impaired by high intensity intermittent exercise. Recent studies have provided a semi-sold meal after exercise and reported that low intensity (brisk walking) or cycling [10,46] did not affect GER after exercise. GER in humans has been shown to be affected by ingested volume and nutrient content [47]. For this reason, during the current study the meal provided was standardised for energy content and volume. It is well known that ingesting protein-rich food immediately after exercise stimulates muscle protein synthesis. Although strenuous continuous exercise delays gastrointestinal function and delivery of nutrients to the circulation, Kashima et al. [48] found that intermittent supramaximal cycling delayed GER of a 300 mL carbohydrate-protein drink when participants consumed the drink 5 and 30-min after exercise compared to a control. This discovery of delayed GER after intense exercise was suggested to be a result of small intestine mucosal damage as a significant increase in intestinal fatty acid-binding protein (I-FABP) was observed in both exercise trials. These findings suggest mucosal damage increases in response to strenuous exercise. Within the current study, a lower DOB for MOD-INT may indicate a potential mechanism, as delayed GER may also result in reduced intestinal absorption, affecting nutrient uptake post-exercise. Further research is needed to understand if small intestine permeability results in a delay in GER after strenuous exercise. However, this theory warrants further investigation as I-FABP was not measured in this study, hence whether increases in I-FABP may have an effect on GER is unclear.

Increased fat oxidation has been suggested to be beneficial for reducing fat mass [49]. The current study found fat oxidation peaked 30-min post ingestion in all conditions compared to baseline values, but a significant increase was only seen during MOD-INT/CON trials. This increase in fat oxidation suggests exercising at 60% VO_2peak_, regardless of the modality of exercise results in an increase in fat metabolism up to 30-min post food consumption. Nevertheless, beyond 60 min fat oxidation reduced, resulting in no differences between exercise conditions in the postprandial period. This corresponds with existing literature, as fasted exercise increases fat metabolism and feeding carbohydrate induces a greater increase in carbohydrate metabolism [46,49]. Furthermore, carbohydrate oxidation significantly peaked during LOW-INT/CON at 60–90 min and, during the 2-h recovery, fat oxidation increased at 90- and 120-min only during the MOD-INT which might suggest carbohydrate oxidation was more heavily relied on in the later stages of MOD-INT compared to other trials. Glucose levels increased mid-exercise during MOD-INT and, regardless of intensity, were lower 30 min after food ingestion when intermittent trials were compared to continuous trials. This suggests that, within a non-endurance trained population, carbohydrate became the primary source for energy during intermittent exercise. This may have resulted in increased muscle and liver glycogen replacement during recovery to maximise muscle glycogen resynthesis, which might account for the change in fat oxidation in the postprandial period. This result should not be misinterpreted, as AUC data for both fat oxidation and carbohydrate oxidation did not show any significant differences. Therefore, the intermittent exercise trial corresponding to similar work output as 60% VO_2peak_ continuous exercise did not significantly increase energy metabolism in the postprandial period.

Our study presents with both strengths and limitations. The main strength of our study is the crossover design, as each modality was matched for power output at a low and moderate intensity. This study is also one of the few that examines GER. A limitation was that we have not accounted for changes in gut-derived hormone data. Previous research has shown that ghrelin regulates GER [50,51] and this may modulate feelings of hunger and EI. Further studies should further examine the differences in post-exercise energy demands after moderate intensity intermittent exercise to understand the causes of why EI was higher, which could have a negative effect on energy balance and possibly relevant exercise outcomes (i.e., weight loss). The manner in which EI were assessed might also be considered as a limitation. When using weighed diet recall it is difficult to minimise mistakes made by the participants and, in addition, the post-exercise diet analysis was undertaken by an experienced member of the research team rather than a qualified dietician, which could have introduced error or bias. Blinding participants from the modality of exercise was impossible and therefore the elevation in EI after the MOD-INT trial was possibly established for the reason that participants thought they should consume more food after the ‘hard’ intermittent exercise. Further work is needed to establish how EI is assessed in the 24–48 h after exercise. Another limitation was the nature of the moderate intensity intermittent exercise when matched for power output to moderate intensity continuous exercise, as the healthy untrained participants within the current study found this session extremely difficult, which resulted in two participants having to withdraw as they were unable to complete MOD-INT trial. This is not unusual as Martins et al. [7] found inactive overweight individuals also struggled when exercise induced an energy expenditure of 250 kCal. This would suggest modality of exercise is an important consideration when designing physical activity sessions.

## 5. Conclusions

We found that despite GER being delayed during MOD-INT, this led to a similar appetite and substrate utilisation response in the short 2-h monitoring period after exercise. However, 24-h EI following MOD-INT was greater than MOD-CON. The mechanisms behind this are unclear as exercise was matched for power output. These findings may have important implications for current exercise prescription guidelines as the modality of exercise appears to have little impact on these markers when performed at a low intensity. It may be important to consider if splitting exercise by completing multiple exercise sessions over the same day would affect appetite regulation, as future studies should aim to develop whether the nature of exercising intermittently lead to increased EI and therefore weight gain.

## Figures and Tables

**Table 1:** Baseline subject characteristics ^1^.

Men (*n* = 12)	
Age, years	30 ± 6
Weight, kg	79.1 ± 0.9
Height, m	1.79 ± 0.08
BMI, kg/m²	24.6 ± 2.0
Body fat, %	17.8 ± 3.9
VO_2_max, mL/kg/min	38 ± 6

^1^ Values are means ± SDs.

**Table 2:** Standardisation measurements for each variable during laboratory visit.

Measurement	LOW-CON	LOW-INT	MOD-CON	MOD-INT
Pre-trial measurements				
Body Mass (KG)	79.3 ± 8.8	79.3 ± 8.9	79.4 ± 8.7	79.6 ± 8.9
Exercise measurements				
Average HR (60-min)	104 ± 16	106 ± 16	139 ± 18	130 ± 17
Work completed (KJ)	277 ± 94	271 ± 73	524 ± 104	518 ± 103
Exercise intensity (W)	77 ± 26 ^†^	222 ± 73 ^†^	145 ± 29 ^†^	423 ± 86 ^†^
Environmental temperature				
During exercise (°C)	20.8 ± 1.1	20.6 ± 0.9	20.7 ± 0.9	21.2 ± 1.1
During recovery (°C)	21.2 ± 1.1	21.0 ± 0.7	21.4 ± 0.8	21.4 ± 0.8
Semi-solid meal				
Time to eat soup (s)	313 ± 166	343 ± 91	405 ± 101	434 ± 91 *

Data are means ± SD. Values are significant *p* < 0.005. * MOD-INT is significantly different from MOD-CON, ^†^ value is significantly different from all other trials.
